# Impact of Standardized Heart Failure Management Center Construction on the Management of Patients With Chronic Heart Failure

**DOI:** 10.1002/clc.70076

**Published:** 2025-01-08

**Authors:** Xiaoxia Guo, Lele Jing, Changlin Zhai, Liang Shen, Huilin Hu

**Affiliations:** ^1^ Department of Cardiology Affiliated Hospital of Jiaxing University Jiaxing China

**Keywords:** chronic heart failure, readmission rate, sodium‐glucose cotransport protein 2 inhibitors, standardized heart failure management center, treatment standardization

## Abstract

**Background:**

Heart failure is extremely harmful to human health and social economics. The purpose of standardized heart failure management center (SHFMC) is to correct the non‐standardization of heart failure treatment.

**Hypothesis:**

SHFMC has a positive impact on the management and prognosis of patients with chronic heart failure (CHF).

**Methods:**

The SHFMC database of Jiaxing First Hospital was retrospectively analyzed. Two hundred sixty‐three patients with CHF who were hospitalized in the cardiovascular medicine department of Jiaxing First Hospital in Zhejiang Province from January 2020 to December 2020 were identified as study subjects. The SHFMC opening day, July 1, 2020, was used as the dividing line around which the patients were divided into Group A (before the completion of SHFMC, *n* = 137) and Group B (after, *n* = 126). The baseline data, treatment standardization, long‐term efficacy, 1‐year all‐cause mortality, and readmission rate of the two groups were compared.

**Results:**

The use of angiotensin receptor enkephalinase inhibitors (ARNIs), β‐blockers (β‐Bs), and sodium‐glucose cotransport protein 2 inhibitors (SGLT2is) increased significantly, and the long‐term outcome, readmission rate, and 1‐year all‐cause mortality of patients improved in group B.

**Conclusions:**

The construction of SHFMC has been associated with consistent improvements in the standardization of CHF treatment, long‐term patient outcomes, 1‐year cumulative survival rates, and readmission rates.

## Graphical abstract

Standardized heart failure management centers have beneficial effects on both hospitalization and long‐term prognosis of heart failure patients.

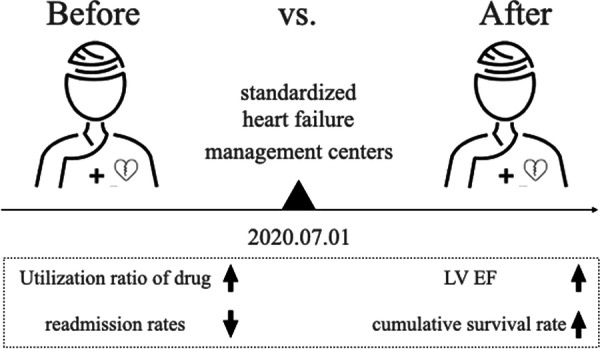

**Abbreviations**
ACEIs/ARBs: angiotensin‐converting enzyme inhibitors/angiotensin II receptor antagonists
ARNIs: angiotensin receptor enkephalinase inhibitors
β‐Bs: β‐blockers
CHF: chronic heart failure
SHFMC: standardized heart failure management center
SGLT2is: sodium‐glucose cotransport protein 2 inhibitors

## 1 Introduction

Chronic heart failure (CHF) is a syndrome of impaired cardiac circulation due to impaired systolic and/or diastolic function of the heart in which venous return is not adequately, resulting in stagnation of blood in the venous system and inadequate perfusion of blood in the arterial system [1, 2]. Chronic heart failure is not an independent disease but the end stage of heart disease and the final battlefield of heart disease treatment. As a severe manifestation or advanced stage of various cardiac diseases, CHF has a high mortality rate [3]. As of 2017, an estimated 64.3 million people worldwide suffered from CHF [4], and with the prevalence of diseases such as hypertension and diabetes and related risk factors, there will be an even larger population of CHF patients in the future.

At present, there are some problems in the management of CHF patients in China [5], including the lack of standardized treatment [6], the lack of long‐term effective management, the differences in the level of diagnosis and treatment between different hospitals or regions [7], and the phenomenon of valuing the “inside” over the “outside” of the hospital. All of these problems may raise readmission rates and the risk of death and reduce the quality of life in patients with CHF. To address these issues, China Cardiovascular Health Alliance and the Cardiovascular Disease Branch of the Chinese Medical Association have jointly decided to construct several standardized heart failure management centers (SHFMCs) across the country. Standardized heart failure management centers hope to improve the overall diagnosis and treatment of CHF in China through the implementation of standardized treatment and long‐term patient follow‐up management based on CHF guidelines. Standardized heart failure management centers have combined data on CHF in China to establish a Chinese‐appropriate treatment model, which should greatly help to improve the quality of patient survival.

SHFMCs consist of two parts, the first of which is the standardized diagnosis and treatment of CHF include the following: (1) standardized diagnostic processes and diagnostic criteria and standardized implementation of them in diagnosis and treatment; (2) improved auxiliary examinations for CHF (including routine examinations + special examinations); and (3) standardized treatment of patients according to guidelines. Second part is long‐term follow‐up of CHF, which includes (1) categorizing and archiving the patient's files in conjunction with their medical records; (2) contacting the patient by telephone from time to time, gaining an understanding of their situation, and providing health education according to the information given; and (3) reminding the patient to review the situation over the course of the telephone follow‐up.

Jiaxing First Hospital opened its SHFMC in July 2020 and established its Heart Failure Center Committee at the same time. Its goals are to integrate medical resources within the hospital, form a multidisciplinary management team, open CHF wards and outpatient clinics, implement hierarchical diagnosis and treatment and a two‐way referral model, standardize the diagnosis and treatment of CHF, improve the overall management of CHF patients, and minimize their risk of rehospitalization and death. This study retrospectively analyzed the data of CHF patients who came to Jiaxing First Hospital on their own 6 months before and 6 months after July 1, 2020, to investigate the impact of the existence of this SHFMC on the standardization of in‐hospital consultation and treatment, long‐term outcomes, and long‐term prognosis of CHF patients.

## 2 Methods

### 2.1 Study Design and Study Population

The study design is shown in Supporting Information. A total of 294 patients with CHF (any etiology) who self‐presented to the Cardiovascular Medicine Department of Jiaxing First Hospital from January 1, 2020 to December 31, 2020 were included in this study. Chronic heart failure was diagnosed with reference to the 2016 European guidelines for the diagnosis and management of acute and CHF [8]. Exclusion criteria: (1) patients unwilling to continue treatment in our hospital for various reasons; (2) patients with chronic kidney disease stage 5: glomerular filtration rate less than 15 mL/min; (3) patients in the end stage of malignancy (based on pathological results); (4) patients with incomplete baseline data; and (5) patients lost to follow‐up.

Thirty‐one noncompliant patients (10.5%) were excluded according to the exclusion criteria. A total of 263 patients were finally included in this study. The launch date of SHFMC at Jiaxing First Hospital was July 1, 2020. Using this time point as the dividing line, patients who presented between January 1, 2020 and June 30, 2020 were included in Group A (*n* = 137), and patients who presented between July 1, 2020 and December 31, 2020 were included in Group B (*n* = 126). Study was in accordance with the Declaration of Helsinki.

### 2.2 General Observation Indicators

General observation indicators included patient age, sex, history of smoking, history of alcohol consumption, history of hypertension, history of hyperlipidemia, history of diabetes mellitus, history of atrial fibrillation, and test results on admission, including NT‐proBNP, eGFR, LV EF, LVEDD, etc.

### 2.3 Special Observation Indicators

#### 2.3.1 Standardization of Drug Treatment

Medications used during hospitalization included angiotensin‐converting enzyme inhibitors/angiotensin II receptor antagonists (ACEIs/ARBs), angiotensin receptor enkephalinase inhibitors (ARNIs), β‐blockers (β‐Bs), oral diuretics (including tab diuretics or thiazide diuretics), aldosterone receptor antagonists (MRAs, mainly spironolactone), anticoagulants (including warfarin and new oral anticoagulants, only patients with atrial fibrillation were counted), digoxin, and sodium‐glucose cotransport protein 2 inhibitors (SGLT2is).

#### 2.3.2 Long‐Term Prognosis

Patients in both groups were followed up at 1 year after discharge, at which time their eGFR, LV EF, LVEDD, readmission rate, all‐cause mortality, cumulative survival, and stroke incidence in patients with atrial fibrillation were recorded.

### 2.4 Statistical Analysis

SPSS 22.0 and GraphPad Prism 7.0 software were used for statistical analysis of the data. Measures conforming to the normal distribution are expressed as mean ± standard deviation, and the t test was used to compare them between groups. The measures with a skewed distribution are expressed as median (quartile spread), and the statistical method was the rank sum test. Count data are expressed as rates or composition ratios, and rates were compared between groups using the chi‐square test or Fisher's exact probability method. Cumulative survival rates were analyzed by drawing K‒M curves. Missing data in follow‐up outcome comparisons were processed by multiple interpolation. Differences were considered statistically significant at *p* < 0.05.

## 3 Results

### 3.1 Baseline Information

The characteristics of the study participants are summarized in Table 1. Patients before the opening of the SHFMC were included in Group A (*n* = 137), and patients after the opening of SHFMC were included in Group B (*n* = 126). The two groups were balanced in terms of age (*p* = 0.637). The mean age was 73.5 years. The proportion of males was 81% in Group A and 72% in Group B (*p* = 0.745). There was also no statistically significant difference in the proportion of smoking and drinking history between the two groups (*p* = 0.088, *p* = 0.085). All patients had systolic blood pressure, diastolic blood pressure and heart rate measured on admission, with no significant differences between the two groups (*p* = 0.750, *p* = 0.071, *p* = 0.919, respectively). All patients were registered with comorbidities on admission, including a history of hypertension, diabetes mellitus, atrial fibrillation, and hyperlipidemia. The difference in the proportion of these comorbidities between the two groups was not statistically significant (*p* = 0.853, *p* = 0.079, *p* = 0.064, *p* = 0.074). Regarding the tests performed on admission of the patients, we found four indicators clearly associated with CHF: NT‐proBNP, eGFR, LV EF, and LVEDD. The results showed that eGFR was somewhat different between the two groups, but the associations of all four indicators were not statistically significant (*p* = 0.157, *p* = 0.072, *p* = 0.555, *p* = 0.541).

**Table 1:** Baseline information of the study participants.

	A group	B group	
	(*n* = 137)	(*n* = 126)	*p*‐value
Age (years)	73.5 ± 12.7	73.5 ± 10.3	0.637
Male, *n* (%)	81 (59.1)	72 (57.1)	0.745
Smoke (%)	24 (17.5)	33 (26.2)	0.088
Drink (%)	8 (5.8)	16 (12.7)	0.085
SBP (mmHg)	116.5 ± 18.5	118.0 ± 14.9	0.750
DBP (mmHg)	65.0 ± 10.0	67.6 ± 11.5	0.071
HR (bpm)	72 (62,80)	70.0 (63,80)	0.919
Comorbidities, *n* (%)			
Hypertension	82 (59.9)	74 (58.7)	0.853
DM	35 (25.5)	21 (16.7)	0.079
AFib	83 (60.6)	62 (49.2)	0.064
Hyperlipemia	19 (14)	9 (7.1)	0.074
Laboratory analyses			
NT‐proBNP (pg/mL)	3650 (1490,7125)	2350 (974,5985)	0.157
eGFR (mL/(min × 1.73 m^2^))	80 (61,97)	86 (65,106)	0.072
LV EF (%)	48 (33,61)	50 (36,60)	0.555
LVEDD (mm)	55 (47,63)	53 (47,59)	0.541

Abbreviations: DBP, diastolic blood pressure; DM, diabetes mellitus; eGFR, estimated glomerular filtration rate; HR, heart rate; LV EF, left ventricular ejection fraction; LVEDD, left ventricular end‐diastolic dimension; SBP, systolic blood pressure.

### 3.2 Etiology of CHF

**Figure 1:**
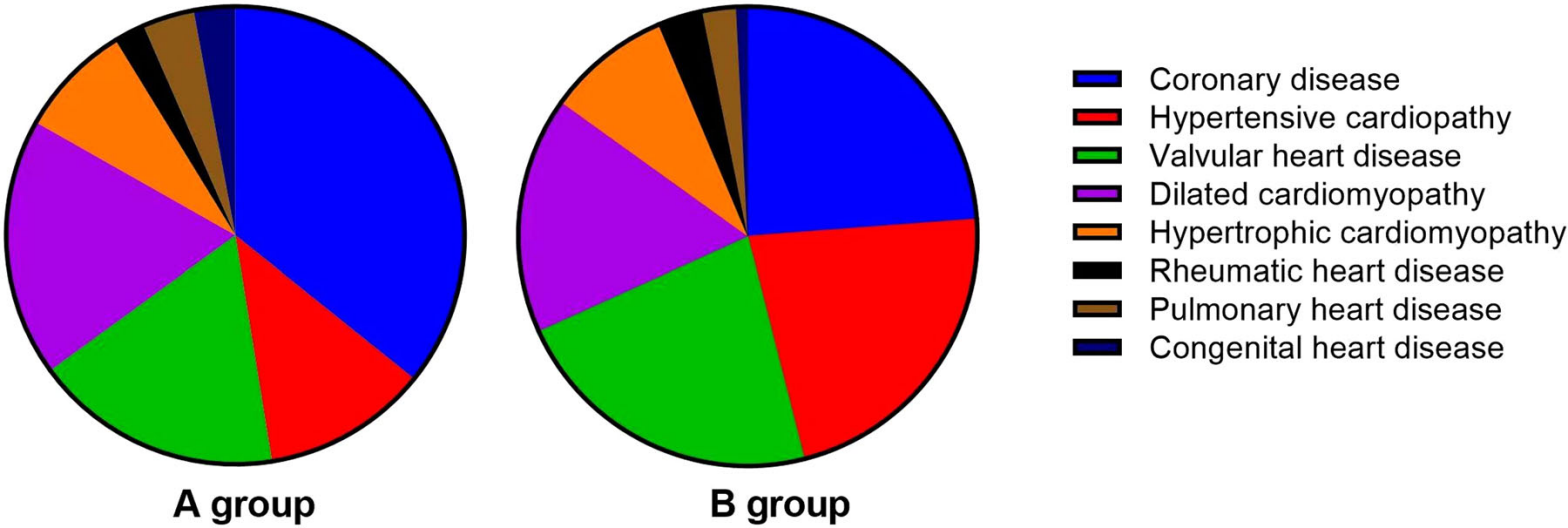
Etiologies of CHF (distribution of causes).

The main causes of CHF include coronary heart disease, hypertensive heart disease, valvular disease, dilated heart disease, hypertrophic cardiomyopathy, rheumatic heart disease, pulmonary heart disease, and congenital heart disease. Comparing the causes of CHF in the two groups (Figure 1), we found that the percentages of patients in Group A with the above causes were 49%, 16%, 24%, 25%, 11%, 3%, 5%, and 4%, and the percentages of patients in Group B were 30%, 28%, 28%, 21%, 11%, 4%, 3%, and 1%, respectively. The difference in distribution between the two groups was not statistically significant (*p* = 0.159).

### 3.3 Medication Standardization

**Figure 2:**
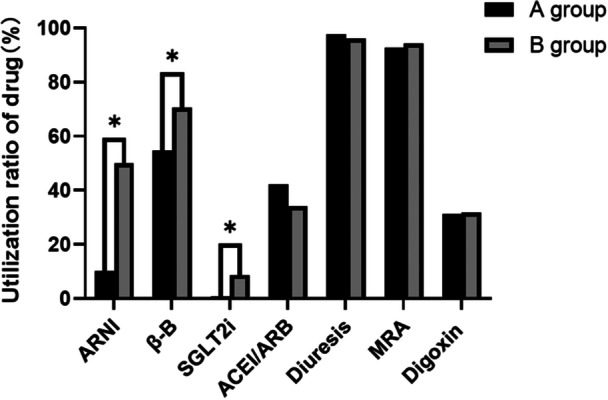
Comparison of utilization rates of different CHF drugs. ARNIs, β‐Bs, SGLT2is, ACEIs/ARBs, diuretics, spironolactone, digoxin. *p < 0.05.

The most important element of an SHFMC is the standardization of the treatment of CHF patients during hospitalization. The guidelines for the treatment of CHF involve drugs such as ACEIs/ARBs, diuretics, spironolactone, digoxin, ARNIs, β‐Bs, and SGLT2is. Of the patients in Group A, 41.9%, 13.1%, 97.8%, 92.7%, and 31.4% took ACEIs/ARBs, diuretics, aldosterone receptor antagonists, and digoxin, respectively, compared to 34.1%, 8.7%, 96%, 94.4%, and 31.7% in Group B (all *p* > 0.05). In contrast, the use of ARNIs, β‐Bs, and SGLT2is increased significantly after the opening of our SHFMC, from 14%, 75%, and 1% to 63%, 89%, and 11%, respectively (all *p* < 0.05, Figure 2). For patients with CHF combined with atrial fibrillation, the use of anticoagulants (including new oral anticoagulants and warfarin) was 32.8% and 38.1% in the two groups, respectively, with no statistically significant difference (*p* > 0.05).

### 3.4 Long‐Term Prognosis Comparison

In addition to standardized treatment, the standardized management component of SHFMCs includes long‐term follow‐up. We followed up both groups for 1 year (both telephone and in‐person follow‐up) and recorded eGFR, LV EF, and LVEDD for all patients when they were revisited 1 year later. The results showed that the mean value of eGFR decreased in both groups compared with the previous value, and there was no difference in the decrease between the two groups (*p* > 0.05). The mean value of LV EF was higher in both groups than 1 year earlier, and the improvement in cardiac function was more pronounced in Group B patients (*p* < 0.05). The mean LVEDD decreased in both groups compared with the previous LVEDD, suggesting that ventricular remodeling was improved in both groups, but patients in Group B benefited more (*p* < 0.05). The readmission rates were 63% and 39% in Groups A and B, respectively, and the all‐cause mortality rates were 23% and 7%, respectively, with statistically significant differences between the two groups (*p* < 0.05). In patients with CHF combined with atrial fibrillation, the stroke rates were 2% and 4% in Groups A and B, respectively, with no statistically significant difference between the two groups (*p* > 0.05, Table 2).

**Table 2:** Comparison of the efficacy and prognosis of patients in both groups.

	A group	B group	*p*‐value
eGFR (mL/(min × 1.73 m^2^))	74 (54,94)	76 (56,102)	0.343
ΔeGFR (mL/(min × 1.73 m^2^))	6 (−5,19)	9 (0,17)	0.329
LV EF (%)	53 (38,62)	58 (47,63)	0.025
LVEDD (mm)	54 (47,61)	51 (47,56)	0.028
Hospital readmission (%)	63 (46)	39 (31)	0.012
All‐cause mortality (%)	23 (16.8)	7 (5.6)	0.004
AFib stroke (%)	2 (2.4)	4 (6.5)	0.227

*Note:* ΔeGFR, absolute value of change in eGFR of patients at 1‐year follow‐up.

### 3.5 Survival Analysis

**Figure 3:**
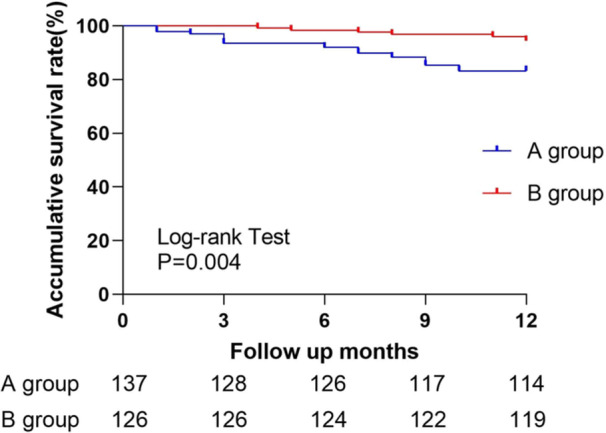
Kaplan–Meier curves for cumulative HF survival.

Kaplan–Meier analysis revealed a significantly higher clinical cumulative survival rate in Group B group than in Group A (*p* = 0.004, log‐rank, Figure 3).

## 4 Discussions

CHF can progress irreversibly, with a precipitous decline in cardiac function after each loss of compensation, culminating in a fatal event [9, 10]. The 2021 update of the Canadian Cardiovascular Society (CCS)/Canadian Heart Failure Society (CHFS) CHF guidelines recommends early initiation of standard therapy and early administration of therapy, which improves the prognosis, as demonstrated by numerous randomized controlled clinical trials. It is titrated every 2–4 weeks to reach the target or maximum tolerated dose after 3–6 months [11]. The PARADIGM‐HF study and the DAPA‐HF study also showed that the more drugs targeting different pathways that were combined, the better the prognosis of the patients [12, 13]. In the DAPA‐HF study, patients on “new quadruple” therapy had a significantly lower event rate than those on triple therapy. A meta‐analysis included 70 RCTs (38 ACEi studies, 21 beta‐blocker studies, 11 aldosterone receptor antagonist studies, with the primary endpoint of all‐cause mortality). The results showed a 12.2% increased risk of mortality in patients where all three classes of HF therapy (ACEIs, β‐Bs, and MRAs) were delayed for 1 year [14]. Furthermore, early in‐hospital initiation of guideline‐directed medical therapy (GDMT) and achieving the target dose is critical to stopping the progression of CHF [15, 16].

By 2021, the number of people with CHF in China will be approximately 12.1 million, and this number will continue to increase, posing a huge economic burden to society [17, 18]. However, the treatment of CHF in China is not standardized, there is a lack of long‐term effective management, there are some differences in the level of diagnosis and treatment between different hospitals or regions, and the phenomenon of emphasizing “in‐hospital” and neglecting “out‐of‐hospital” has caused shortcomings in long‐term care. The construction of SHFMCs aims to solve these problems. On May 21, 2021, the SHFMC of Jiaxing First Hospital received its on‐site certification from the expert team at the headquarters of the China Heart Failure Center and was highly recognized by the expert team. The hospital's national SHFMC accreditation was officially approved on July 1.

To investigate the effect of SHFMC construction on HF management, we enrolled 263 CHF patients in this study, the inpatients before SHFMC construction being included in Group A (*n* = 137) and those after SHFMC construction in Group B (*n* = 126). Results showed that the proportion of ARNIs, β‐Bs, and SGLT2is used by patients increased significantly after SHFMC construction. Comparing the data of the two groups at the follow‐up visit 1 year after discharge, we found that LV EF was higher and LVEDD was lower in both groups than they were at admission. The readmission rate and all‐cause mortality rate of patients in Group B were significantly lower than those in Group A. In patients with CHF combined with atrial fibrillation, the proportion of anticoagulant use and the incidence of stroke in atrial fibrillation did not differ between the two groups. Finally, K‒M curves showed that the cumulative survival rate at 1 year of discharge was significantly higher in Group B patients than in Group A patients.

In two recent CHF guidelines [19, 20], the clinical benefit of the new quadruple anti‐cardiac failure drugs (ARNIs/ACEIs/ARBs, β‐Bs, MRAs, SGLT2is) has been well established. Guideline‐directed medical therapy has been advocated for widespread adoption to improve outcomes, but there is an inherent tension between GDMT and polypharmacy. The risks quantified by clinical trial data may be underestimated because randomized controlled trials have traditionally excluded populations most vulnerable to adverse drug events, such as elderly and multimorbid patients [21, 22]. Therefore, clinicians need to conduct an individualized risk–benefit assessment for each patient and always consider the risk of adverse events when prescribing medications (even those that appear in clinical practice guidelines).

Several limitations of this study should be considered. First, we did not discuss the different types of CHF (including CHF with reduced ejection fraction, CHF with intermediate ejection fraction, CHF with preserved ejection fraction, and CHF with improved ejection fraction) separately. Second, the normative use of intravenous diuretics, intravenous vasodilators, and intravenous cardiac drugs was not counted in this study, and only the use of some oral anti‐heart failure drugs was counted. Third, the follow‐up period was short. In the future, we will conduct long‐term follow‐up of these patients to further verify the impact of SHFMC construction on the prognosis of CHF patients.

## 5 Conclusions

Our study confirms that the construction of the Jiaxing SHFMC was associated with the standardization of drug therapy and consistent improvement in the long‐term prognosis of CHF patients. The construction of other SHFMCs is expected to have similar benefits elsewhere in China.

## Author Contributions

All authors contributed to the article and approved the submitted version.

## Ethics Statement

The authors have nothing to report.

## Consent

The authors have nothing to report.

## Conflicts of Interest

The authors declare no conflicts of interest.

## Supporting information

Supporting information.

Supporting information.

## Data Availability

The authors confirm that the data supporting the findings of this study are available within the article.
