# Supplementary figures and images for: Impact of Standardized Heart Failure Management Center Construction on the Management of Patients With Chronic Heart Failure
Source: Clin Cardiol. 2025 Jan 8;48(1):e70076. doi: 10.1002/clc.70076 (PMC11711211; doi:10.1002/clc.70076)

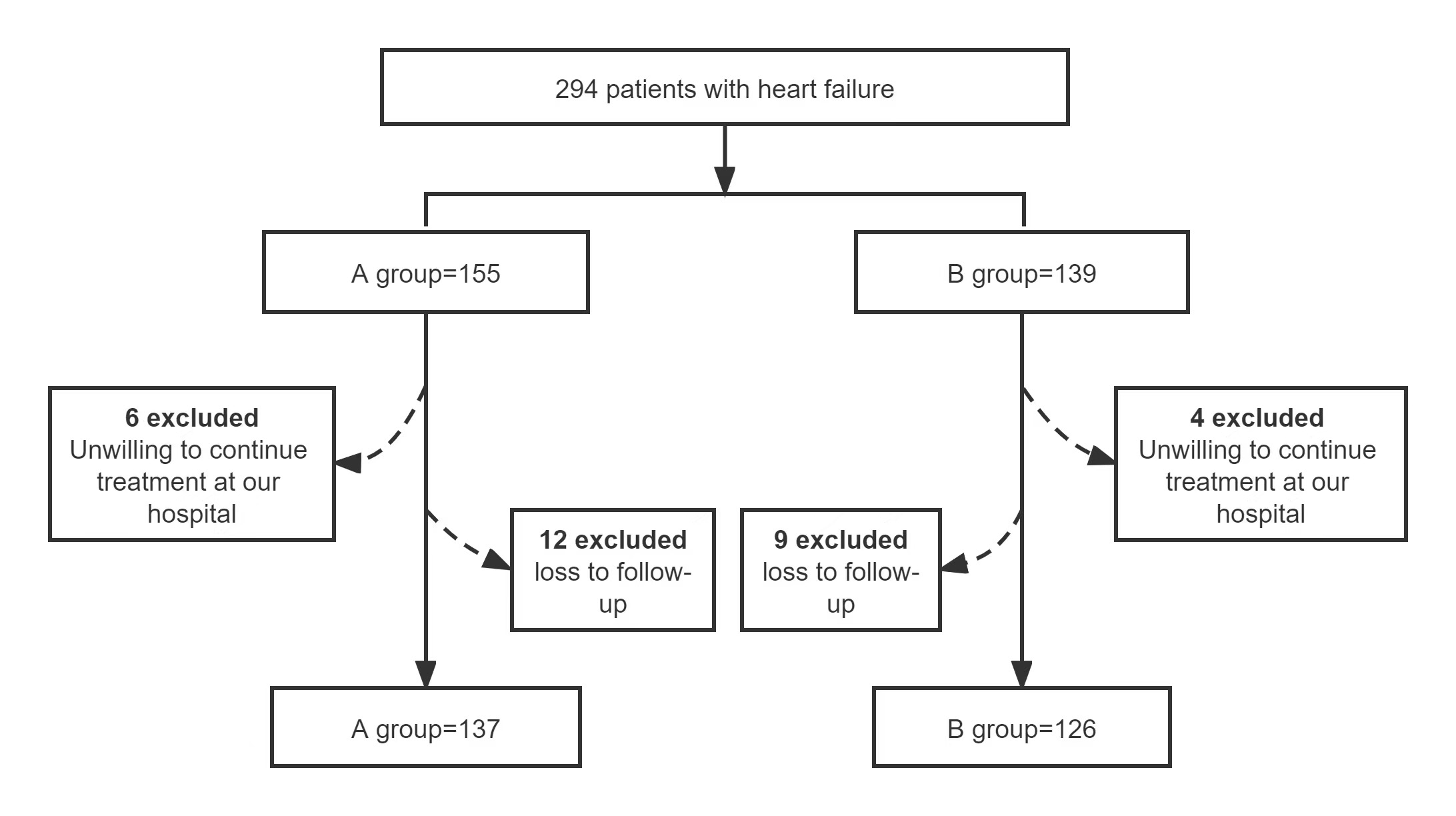

Supplement: Supplementary file 1 — Supporting information. [file CLC-48-e70076-s002.jpg]
